# Effect of acute altitude exposure on physiological parameters and glucose metabolism in healthy lowland Peruvians

**DOI:** 10.12688/f1000research.134346.1

**Published:** 2023-06-21

**Authors:** Lissett Jeanette Fernandez - Rodriguez, Victor Hugo Bardales-Zuta, Gustavo Adolfo Vásquez-Tirado, Carlos Avalos Alvarado, Eva J Schaefer, Julio Hilario-Vargas

**Affiliations:** 1 Physiology Laboratory, School of Medicine, Universidad Privada Antenor Orrego, Trujillo, La Libertad, Peru; 2 Albert Ludwig University of Freiburg, Freiburg, Germany; 3 Department of Physiology, School of Medicine, Universidad Nacional de Trujillo, Trujillo, La Libertad, Peru

**Keywords:** altitude sickness, hypoxia, insulin, C-peptide, cortisol

## Abstract

Background: High altitude exposure triggers a series of physiological changes to maintain homeostasis. Although longer-term (days to years) acclimatization processes are well studied, less is known about the physiological changes upon rapid ascent. We took advantage of Peru’s geography to measure the first physiological changes following rapid transport from a low to a high-altitude environment among lowlanders.

Methods: Blood glucose, insulin, C-peptide, and salivary cortisol among healthy lowland Peruvians were measured before and after glucose ingestion at 40 m and upon arrival at 3470 m. Resting heart rate, blood oxygen saturation, and blood pressure were also monitored.

Results: At high altitude, we find a significant ( *p*<0.05) increase in heart rate and a decrease in blood oxygen saturation and salivary cortisol. Additionally, baseline levels of blood glucose, plasma C-peptide, and cortisol were reduced ( *p*<0.05). Blood glucose, plasma insulin, and plasma C-peptide returned to baseline or below faster at high altitude after glucose ingestion.

Conclusions: Although many overlapping environmental and physiological factors are present in the high-altitude environment, the first steps of acclimatization in this population appear to be caused by increased energy expenditure and glucose metabolism to maintain oxygen homeostasis until the longer-term acclimatization mechanisms become more significant.

## Introduction

Humans have been traveling between high and low altitudes since prehistory. Today, it has been estimated that approximately 40 million lowlanders visit a high-altitude environment, which is marked by increased wind velocity, solar radiation, topographic variation, and temperature variability, as well as lower humidity, average temperature, and atmospheric pressure ( Luks *et al.* 2021). To maintain homeostasis at high altitude, the human body has developed short-term, mid-term, and long-term coping mechanisms.

The altitude acclimatization process has already been studied extensively, providing insight into various altitude-coping physiological processes and to better treat and prevent altitude sickness ( Parati *et al*. 2018, Narvaez-Guerra *et al.* 2018, Torlasco *et al.* 2020, Luks *et al.* 2021). These studies reveal that the acclimatization process involves both persistent and transient physiological changes. However, we are unaware of any published reports that describe the very first physiological changes (<1 h) upon arrival in a real high-altitude environment. One study that analysed physiological changes in a hypobaric chamber suggested that immediate physiological changes may differ from longer-term acclimatization ( Woolcott *et al.* 2015), leading us to hypothesize that short-term physiological compensation to high altitude may differ from longer-term acclimatization mechanisms.

Therefore, to better understand the first steps of altitude acclimatization, we measured blood pressure, heart rate, oxygen saturation, and pulse rate in 15 young healthy lowland participants near sea level and immediately after rapidly ascending to 3470 meters above sea level (masl). Furthermore, blood glucose, plasma c-reactive peptide, plasma insulin, and salivary cortisol were measured as a fasting baseline and after ingestion of a glucose bolus at both low and high altitude. These parameters have been previously measured in mid- to longer- term altitude studies allowing for direct comparison between shorter and longer-term altitude acclimatization ( Woolcott *et al.* 2015, Parati *et al*. 2018, Narvaez-Guerra *et al.* 2018, Torlasco *et al.* 2020, Luks *et al.* 2021). Therefore, we hypothesized that there may be physiological differences between immediate and longer-term altitude acclimatisation.

## Methods

This study was approved by the Research Office of Universidad Privada Antenor Orrego (UPAO), resolution number 0372-2014-R-UPAO. Research office approval requires ethical review of the project. Physiology and endocrinology students of UPAO were invited to participate voluntarily in the study and provided written informed consent. As this is a convenience sample study, caution should be taken in applying these results to the general population. This research was not preregistered at an independent registry. Neither participation nor non-participation had any effect on the academic records of the students. Potential participants were excluded if they had conditions that affected the cardiovascular system, glucose metabolism or the hypothalamic-pituitary-adrenal axis, used tobacco or caffeine, regularly participated in strenuous activity, took drugs that affected the hypothalamic-pituitary-adrenal axis (such as steroids, corticosteroids, growth hormone, thyroid hormone, vasopressin, or dopamine) or if they travelled to high altitude locations less than a year before the study. Only participants for which an entire dataset was generated was included in the study. Participants self-reported their genders.

At the UPAO physiology laboratory in Trujillo, Peru (40 masl), fasting (>8 h) participants were subjected to blood oxygen saturation, resting pulse rate, weight, height, waist-hip ratio and fasting blood glucose, plasma insulin, plasma C-peptide and salivary cortisol measurements. The participants then drank an aqueous solution containing 70 g of anhydrous glucose (Dropaksa, Trujillo), dissolved in 300 mL of water. Blood glucose, plasma insulin, plasma C-peptide, and salivary cortisol measurements were taken 30, 60, 90, and 120 min after drinking the glucose solution.

Several days later, fasting (>8 h) participants travelled together from Trujillo to Salpo, Perú (3470 masl) and the same tests were completed upon arrival, except for the anthropometric measurements. Blood samples were centrifuged and cooled. Plasma and saliva were processed in the UPAO physiology laboratories and at the National University of Trujillo; blood glucose was measured on-site.

The tests occurred at approximately 9:00 AM at both locations. As much as possible, the participants maintained their daily schedules and wake-up times for both study days. All data acquisition took place in December 2014.

### Procedures

All equipment was used and calibrated according to the manufacturer’s instructions at high and low altitude, where applicable. Blood pressure, oxygen saturation, and pulse rate were measured with a digital blood pressure monitor CH-452 Lot 906 (Citizen Systems Japan Co., Ltd) and a pulse oximeter MF-415 (More Fitness), respectively. Since both monitors also measure pulse rate, the average reading was used. Blood glucose was measured using the Accu-Chek glucose meter (Roche), which was appropriately calibrated at both study locations. Plasma C-peptide, plasma insulin, and salivary cortisol were tested with kits from Gateways Medical (Catalogue Numbers: 80-CPTHU-E01.1, 80-INSHU-E01, 11-CORHU-E01- SLV) according to manufacturer instructions. An ELISA plate reader (Bio-Rad Laboratories Inc.) was also used to process the samples.

### Statistics

A two-sample t-test with Bonferroni correction was applied to search for differences between men and women participants for all data sets taken. To compare corresponding data taken in Trujillo vs. Salpo and baseline vs. timepoint results, a Shapiro-Wilk test was performed to test for normality. If the data set was normally distributed, a paired t-test was performed, if not, a Wilcoxon test was used to account for statistical bias. A result was considered significant if *p*<0.05.

## Results

Of the 254 medical students enrolled in UPAO at the time, 71 were asked to participate in the study. Of these, 15 participants (11 females and 4 males) between 17 and 23 years of age met the acceptance criteria and completed the study. Table 1 reports the anthropometric data of the study participants taken in Trujillo. The only statistically significant difference found between sexes using a two-sample t-test with Bonferroni correction was height, *p*<0.0001, with men taller than women.

**Table 1.** Anthropometric measurements of participants. Anthropometric measurements of study participants taken in Trujillo, significant differences between men and women are indicated with an asterisk. Values are means ± SD; (n=15).

	Women	Men	Combined
Age (years)	19.4±1.7	19.0±0.8	19.3±1.5
Weight (kg)	53.6±5.0	63.6±9.4	56.3±9.17
Height (cm)	155±2.3	169±5.3	159.1±7.1*
BMI (kg/m ^2^)	22.2±1.9	222.1±1.9	22.2±1.9
Waist (cm)	70.3±3.8	78.2±7.5	72.4±5.6
Hip (cm)	86.0±5.6	89.2±12.2	86.9±6.8
Waist-Hip ratio	0.82±0.07	0.88±0.04	0.84±0.07

**Figure 1.**
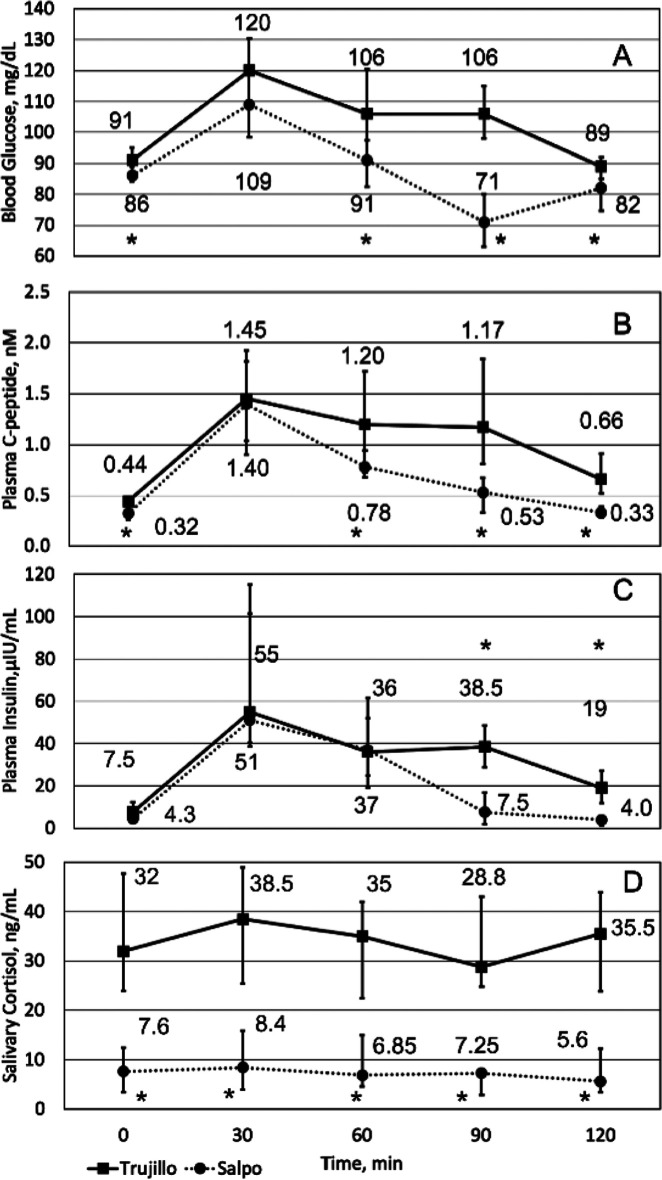
Glucose tolerance at different elevations. Median blood glucose (A), plasma insulin (B), and plasma C-peptide (C), and salivary cortisol (D) for Trujillo (squares, solid line) and Salpo (circles, dotted line) before (0 min) and after (30-120 min) ingestion of 70 g of glucose. Whiskers represent the interquartile range of the 15 participants. Significant differences ( *p*<0.05) between Trujillo and Salpo are indicated with asterisks.

Table 2 and Figure 1 (0-minute time point) record the physiological baseline measurements in Trujillo and Salpo. Oxygen saturation, blood glucose, plasma C-peptide, and salivary cortisol were significantly lower in Salpo than in Trujillo, while heart rate was higher in Salpo ( *p*=0.02 for C-peptide; *p*<0.004 for other measurements).

**Table 2.** Physiological measurements of participants. Oxygen saturation (O _2_SAT), pulse rate, systolic (SBP) and diastolic (DBP) blood pressure measurements of study participants in Trujillo and Salpo. Values are means ± SD. In the rightmost column, the *p*-value of the paired t-test is listed, which indicates whether a statistically significant difference was found between Trujillo and Salpo.

	Trujillo	Salpo	*p*
O _2_SAT (%)	98.8±0.5	88.1±3.7	<0.0001
Pulse rate, min ^-1^	77.3±11	91.8±10	<0.0001
SBP (mmHg)	110±11	113±12	0.47
DBP (mmHg)	75.0±9.7	76.9±6.6	0.34

The baseline and sequential measurements taken after consuming 70 g glucose reveal significant differences in glucose metabolism after rapid ascent ( Figure 1). Insulin, glucose, and C-peptide levels rose to approximately the same concentrations in both locations but decreased more quickly in Salpo, resulting in statistically significant lower values between datasets. Throughout the time course, salivary cortisol was significantly lower in Salpo ( *p*<0.0008).

During the time course some notable differences occurred ( Figure 1): (1) blood glucose at 90 and 120 minutes was lower than baseline in Salpo ( *p*<0.002), while it was above or indistinguishable from baseline in Trujillo; (2) C-peptide remained significantly above baseline throughout the time course in Trujillo ( *p*<0.004), but was indistinguishable from baseline at 120 minutes in Salpo; (3) insulin became indistinguishable from baseline after 90 minutes in Salpo, but remained above baseline throughout the entire time course in Trujillo ( *p*<0.0008); and (4) salivary cortisol did not show statistically significant changes throughout the time course for both Trujillo and Salpo. Statistical analysis using the t-test or the Wilcoxon test gave nearly identical results regardless of normality.

## Discussion

Eight physiological parameters were measured in 15 healthy Peruvian students at low-altitude Trujillo (40 masl) and high-altitude Salpo (3470 masl), taking advantage of Peru’s geography, where high altitude regions are accessible from the coast approximately 3 hours by road. Unlike other studies that take initial measurements after a stepwise ascent or within the first days of arrival, this study tested unacclimatized lowlanders within an hour of arrival at high altitude, thus allowing greater understanding of the first physiological changes associated with rapid ascent to a real high-altitude environment from sea level. Furthermore, the participants were driven to Salpo, which controls for physical exertion. To control for daily variations in certain physiological parameters, measurements were taken at approximately the same time of day and participants maintained their daily routine as much as possible. Since only height was statistically different between men and women for all parameters tested, male and female participants were treated as one group.

Combining the results ( Table 2, Figure 1), we can outline the physiological changes that take place immediately upon arrival at high altitude and propose the underlying mechanisms. Because partial oxygen pressure decreases with increasing altitude, blood oxygen saturation also decreases, which stimulates the sympathetic nervous system and peripheral chemoreceptors, increasing heart and breathing (respiratory) rates to maintain acceptable blood oxygen levels, which has already been reported ( Luks *et al*. 2021). This causes an increase in energy demand. Furthermore, glucose metabolism becomes favoured over fatty acid oxidation at high altitude ( Braun 2008). As a result, glucose consumption increases, causing increased postprandial elimination of glucose and a faster decrease in insulin and C-peptide. This result closely resembles a finding using prompt *simulated* altitude exposure ( Woolcott *et al.* 2015).

Altitude and cortisol seem to be positively correlated, especially at very high altitude, but not so much at moderate or high altitude ( Woods *et al.* 2012). This correlation has been used to partly explain the increase in glycemia during subacute altitude exposure ( Woolcott *et al.* 2015, Koufakis *et al.* 2019). In our study, salivary cortisol was significantly higher in Trujillo than in Salpo and was insensitive to glucose ingestion. This may partly explain the lower baseline glucose, improved glucose tolerance, and no significant increase in blood pressure in Salpo. Since cortisol is also associated with mid- and long-term stress, we cannot rule out that the lower cortisol observed here may be a result of the short respite from their academic studies the students experienced on their day in Salpo, but this is not easily controlled because a high-altitude town differs greatly from a large provincial city. However, this observation is likely to be relevant for other high-altitude travellers because such trips are often made for recreation.

The physiological changes observed here are likely transient and only last several hours, as other studies involving longer-term altitude exposure have shown that within the first day or two the body compensates with above normal glycemia in response to sympathetic stimulation and increased cortisol, which then decreases after the first week at high altitude. Longer-term sojourns tend to improve glucose tolerance beyond the corresponding low-altitude values. Indeed, high-altitude residents tend to have healthier glycemia and glucose tolerance than lowlanders. However, other factors associated with people living at altitude, such as degree of physical activity, food availability, and genetics and ethnicity, are also important in explaining physiological differences between highlanders and lowlanders ( Braun 2008, Woods *et al.* 2012, Woolcott *et al.* 2015, Parati *et al.* 2018, Narvaez-Guerra *et al.* 2018, Koufakis *et al.* 2019, Heggie 2019, Torlasco *et al.* 2020, Luks *et al.* 2021). Therefore, additional work, likely with more participants that better reflect the demographics of high-altitude visitors, will be necessary to confirm these results.

Nonetheless, these findings may be important in understanding the first stages of altitude sickness. This work may also be useful for people with type-1 diabetes or other people with glucose-metabolism disorders who visit high altitude due to the lower insulin levels observed among participants upon arrival, higher insulin levels within a few days of arrival, which then decrease ( Richards and Hillebrandt 2013).

In this work, we have observed some aspects of the very first physiological changes that take place upon arrival in a real high-altitude environment among healthy young people. These early changes seem to centre around increased glycolysis to maintain oxygen homeostasis without high glycemia or elevated cortisol. These effects are likely transient, as the longer-term processes associated with altitude acclimatization become more important hours and days after arrival.

## Data Availability

Figshare: Trujillo-Salpo Dataset. DOI: 10.6084/m9.figshare.22685278 This project contains the following underlying data:
• dataset060123.txt (Supplementary dataset for the article “Effect of acute altitude exposure on physiological parameters and glucose metabolism in healthy lowland Peruvians”.) Data are available under the terms of the Creative Commons Zero “No rights reserved” data waiver (CC0 1.0 Public domain dedication). Figshare: STROBE checklist for “Effect of acute altitude exposure on physiological parameters and glucose metabolism in healthy lowland Peruvians”. DOI: 10.6084/m9.figshare.22687624 Data are available under the terms of the Creative Commons Zero “No rights reserved” data waiver (CC0 1.0 Public domain dedication).
