# Supplementary material for: Effect of acute altitude exposure on physiological parameters and glucose metabolism in healthy lowland Peruvians
Source: F1000Res. 2023 Nov 8;12:724. Originally published 2023 Jun 21. [Version 2] doi: 10.12688/f1000research.134346.2 (PMC10654490; doi:10.12688/f1000research.134346.2)
Supplement: Peer review history

# Reviewer report: Reviewer response for version 2

**DOI:** 10.5256/f1000research.158350.r221703

**Published:** 2023-11-16

**Authors:** Robert K Szymczak

**Affiliations:** 1 Department of Emergency Medicine, Faculty of Health Sciences, Medical University of Gdańsk, Gdańsk, Poland

No further improvements are needed.

Is the work clearly and accurately presented and does it cite the current literature?

Partly

If applicable, is the statistical analysis and its interpretation appropriate?

Yes

Are all the source data underlying the results available to ensure full reproducibility?

Yes

Is the study design appropriate and is the work technically sound?

Yes

Are the conclusions drawn adequately supported by the results?

Yes

Are sufficient details of methods and analysis provided to allow replication by others?

Yes

Reviewer Expertise:

Emergency medicine, high altitude medicine

I confirm that I have read this submission and believe that I have an appropriate level of expertise to confirm that it is of an acceptable scientific standard.

# Reviewer report: Reviewer response for version 1

**DOI:** 10.5256/f1000research.147395.r205874

**Published:** 2023-10-10

**Authors:** Robert K Szymczak

**Affiliations:** 1 Department of Emergency Medicine, Faculty of Health Sciences, Medical University of Gdańsk, Gdańsk, Poland

The main goal of the presented article is to expand knowledge about the first steps of altitude acclimatization  - during the first hours of acute high altitude exposure of not acclimatized lowlanders. The research included measurements of blood glucose, insulin, C-peptide and salivary cortisol at sea level and at altitude of 3470m before and after glucose ingestion. The study found that baseline levels of glucose, C-peptide and cortisol were reduced at high altitude. Another result was that the blood glucose, plasma insulin and C-peptide returned to baseline or below faster at high altitude than at sea level after ingestion of glucose. Authors concluded that above results can be explained by increased energy expenditure and glucose metabolism to maintain oxygen homeostasis at high altitude.

 In my opinion, the article is publishable, but needs minor improvements in the discussion section.

 Authors should expand discussion section and compare their results to those presented by other authors. There are few works in which cortisol levels, similarly to results presented in the manuscript, were lower at high altitude in comparison to seal level:

- Woods et al. The cortisol response to hypobaric hypoxia at rest and post-exercise, Horm Metab Res, 2012 Apr;44(4):302-5. doi: 10.1055/s-0032-1304322.
- Von Wolff et al. Adrenal, thyroid and gonadal axes are affected at high altitude, Endocr Connect, 2018 Oct 1;7(10):1081-1089. doi: 10.1530/EC-18-0242.

There are also works in which glucose and insulin levels at high altitude were analyzed, were similarly to results of the presented manuscript, the baseline levels of blood glucose and plasma insulin were decreased at high altitude.

- Stock at al. Effects of exercise, altitude, and food on blood hormone and metabolite levels, J Appl Physiol Respir Environ Exerc Physiol 1978 Sep;45(3):350-4. doi: 10.1152/jappl.1978.45.3.350.

Table 1. Value of BMI for Men has a wrong value 222.1. It has to be changed.

Is the work clearly and accurately presented and does it cite the current literature?

Partly

If applicable, is the statistical analysis and its interpretation appropriate?

Yes

Are all the source data underlying the results available to ensure full reproducibility?

Yes

Is the study design appropriate and is the work technically sound?

Yes

Are the conclusions drawn adequately supported by the results?

Yes

Are sufficient details of methods and analysis provided to allow replication by others?

Yes

Reviewer Expertise:

emergency medicine, high altitude medicine

I confirm that I have read this submission and believe that I have an appropriate level of expertise to confirm that it is of an acceptable scientific standard, however I have significant reservations, as outlined above.

## References

[1] : The cortisol response to hypobaric hypoxia at rest and post-exercise. *Horm Metab Res* .2012;44(4) : 10.1055/s-0032-1304322 302-5 10.1055/s-0032-1304322 22368038

[2] : Adrenal, thyroid and gonadal axes are affected at high altitude. *Endocr Connect* .2018;7(10) : 10.1530/EC-18-0242 1081-1089 10.1530/EC-18-0242 30352395PMC6198189

[3] : Effects of exercise, altitude, and food on blood hormone and metabolite levels. *J Appl Physiol Respir Environ Exerc Physiol* .1978;45(3) : 10.1152/jappl.1978.45.3.350 350-4 10.1152/jappl.1978.45.3.350 701119

# Response

**Published:** 2023-10-30

**Authors:** Lissett Fernández

**Affiliations:** Physiology Laboratory, Universidad Privada Antenor Orrego, Trujillo, La Libertad, Peru

The authors expanded the discussion by approximately 400 words and included the suggested references regarding cortisol and glucose levels at high altitude. 

 The error in Table 1 was corrected.

# Reviewer report: Reviewer response for version 1

**DOI:** 10.5256/f1000research.147395.r202144

**Published:** 2023-08-31

**Authors:** Takayuki Nishimura

**Affiliations:** 1 Department of Human Life Design and Science, Faculty of Design, Kyushu University, Fukuoka, Japan

This paper is overall well written and there are no particular problems. Short-term adaptation to high altitudes is an important issue and is also relevant to prevention of AMS. There is few studies that tested glucose metabolism in high-altitude, and this study provides important findings. However, it may be better to perform a more detailed analysis, for example, a correlation analysis of the relationship between glucose metabolic function and SpO2 and cortisol. Decreasing cortisol in high altitude is interesting but difficult to understand. Please refer this article, it may help your study.

 Bouissou, P. et al. Effect of beta-adrenoceptor blockade on renin-aldosterone and alpha-ANF during exercise at altitude. J. Appl. Physiol. 67, 141–146 (1989).

 Minor point;  the BMI description for men in Table 1 is incorrect.

Is the work clearly and accurately presented and does it cite the current literature?

Yes

If applicable, is the statistical analysis and its interpretation appropriate?

Partly

Are all the source data underlying the results available to ensure full reproducibility?

Yes

Is the study design appropriate and is the work technically sound?

Yes

Are the conclusions drawn adequately supported by the results?

Yes

Are sufficient details of methods and analysis provided to allow replication by others?

Yes

Reviewer Expertise:

Anthropology

I confirm that I have read this submission and believe that I have an appropriate level of expertise to confirm that it is of an acceptable scientific standard.

## References

[1] : Effect of beta-adrenoceptor blockade on renin-aldosterone and alpha-ANF during exercise at altitude. *J Appl Physiol (1985)* .1989;67(1) : 10.1152/jappl.1989.67.1.141 141-6 10.1152/jappl.1989.67.1.141 2527226

# Response

**Published:** 2023-10-30

**Authors:** Lissett Fernández

**Affiliations:** Physiology Laboratory, Universidad Privada Antenor Orrego, Trujillo, La Libertad, Peru

A correlation analysis between the parameters mentioned was completed and included in the updated manuscript. The correlation was done for the changes in oxygen saturation, blood glucose, C-peptide, insulin and cortisol with each participant representing a datapoint. The analysis revealed that there was no correlation between parameters. For instance, we found that the change in oxygen saturation did not predict the change in blood glucose for the participants tested. Furthermore, low-altitude values for blood parameters did not predict their corresponding high-altitude values.

 The resting initial values for blood oxygenation, glucose, c-peptide, insulin, and cortisol were also tested pairwise to search for correlations. Some moderate correlations were found between glucose, c-peptide, and insulin, but cortisol had only weak correlation (r squared < 0.25) against the other parameters.

 We also expanded the discussion by approximately 400 words, included a reference to the mentioned paper and corrected the error in Table 1.
